# Parallel Spinal Pathways for Transmitting Reflexive and Affective Dimensions of Nocifensive Behaviors Evoked by Selective Activation of the Mas-Related G Protein-Coupled Receptor D-Positive and Transient Receptor Potential Vanilloid 1-Positive Subsets of Nociceptors

**DOI:** 10.3389/fncel.2022.910670

**Published:** 2022-05-24

**Authors:** Liang-Biao Wang, Xiao-Jing Su, Qiao-Feng Wu, Xiang Xu, Xin-Yue Wang, Mo Chen, Jia-Reng Ye, Abasi Maimaitiabula, Xiao-Qing Liu, Wen Sun, Yan Zhang

**Affiliations:** ^1^Stroke Center & Department of Neurology, The First Affiliated Hospital of USTC, Division of Life Sciences and Medicine, University of Science and Technology of China, Hefei, China; ^2^Division of Life Sciences and Medicine, University of Science and Technology of China, Hefei, China

**Keywords:** Mrgprd, TRPV1, nociceptor, reflexive, affective, nerve injury

## Abstract

The high incidence of treatment-resistant pain calls for the urgent preclinical translation of new analgesics. Understanding the behavioral readout of pain in animals is crucial for efficacy evaluation when developing novel analgesics. Mas-related G protein-coupled receptor D-positive (Mrgprd^+^) and transient receptor potential vanilloid 1-positive (TRPV1^+^) sensory neurons are two major non-overlapping subpopulations of C-fiber nociceptors. Their activation has been reported to provoke diverse nocifensive behaviors. However, what kind of behavior reliably represents subjectively conscious pain perception needs to be revisited. Here, we generated transgenic mice in which Mrgprd^+^ or TRPV1^+^ sensory neurons specifically express channelrhodopsin-2 (ChR2). Under physiological conditions, optogenetic activation of hindpaw Mrgprd^+^ afferents evoked reflexive behaviors (lifting, etc.), but failed to produce aversion. In contrast, TRPV1^+^ afferents activation evoked marked reflexive behaviors and affective responses (licking, etc.), as well as robust aversion. Under neuropathic pain conditions induced by spared nerve injury (SNI), affective behaviors and avoidance can be elicited by Mrgprd^+^ afferents excitation. Mechanistically, spinal cord-lateral parabrachial nucleus (lPBN) projecting neurons in superficial layers (lamina I–II_*o*_) were activated by TRPV1^+^ nociceptors in naïve conditions or by Mrgprd^+^ nociceptors after SNI, whereas only deep spinal cord neurons were activated by Mrgprd^+^ nociceptors in naïve conditions. Moreover, the excitatory inputs from Mrgprd^+^ afferents to neurons within inner lamina II (II_*i*_) are partially gated under normal conditions. Altogether, we conclude that optogenetic activation of the adult Mrgprd^+^ nociceptors drives non-pain-like reflexive behaviors *via* the deep spinal cord pathway under physiological conditions and drives pain-like affective behaviors *via* superficial spinal cord pathway under pathological conditions. The distinct spinal pathway transmitting different forms of nocifensive behaviors provides different therapeutic targets. Moreover, this study appeals to the rational evaluation of preclinical analgesic efficacy by using comprehensive and suitable behavioral assays, as well as by assessing neural activity in the two distinct pathways.

## Introduction

Chronic pain affects approximately 20% of the human population on a global scale and results in a heavy socioeconomic burden (Vos et al., 2015; Dahlhamer et al., 2018). Opioid abuse and addiction (Compton and Volkow, 2006) compel the urgency to develop new analgesic drugs. To date, the clinical translation from basic science to effective analgesics is poor. One of the challenges is how well behavioral assays in pain assessment reliably reflect the pain experience in humans (Borsook et al., 2014; Tuttle et al., 2015; Mogil, 2018; Ma, 2022). In response to stimuli that potentially or actually cause tissue injury, animals will display a series of defensive responses, including reflexive and affective behaviors. In animal studies lacking a report of an individual experience as pain, people usually treated reflexive behaviors such as withdrawal motor reflex as an indication of pain. However, a study showed that paw withdrawal in *Na_*v*_1.8-ChR2* mice following transdermal light stimulation was resistant to analgesics, such as morphine and pregabalin, while affective responses such as licking, jumping and vocalization were sensitive, suggesting that reflexive behaviors may not be necessary for the manifestation of pain (Daou et al., 2013). A recent study also challenged the validity of using reflexive defensive responses to measure sustained pain (Huang et al., 2019). Understanding the behavioral readout of pain in mice is essential for preclinical evaluation of analgesic efficacy to promote drug discovery.

In 2020, the International Association for the Study of Pain (IASP) defined pain as “an unpleasant sensory and emotional experience associated with, or resembling that associated with, actual or potential tissue damage” (Raja et al., 2020) and expanded that “Pain and nociception are different phenomena. Pain cannot be inferred solely from activity in sensory neurons.” In other words, activation of nociceptors does not always produce pain. Mrgprd^+^ and TRPV1^+^ cutaneous fibers are two major non-overlapping groups of nociceptive afferents expressing isolectin B4 (IB4) and calcitonin gene-related peptide (CGRP), respectively (Zylka et al., 2005; Cavanaugh et al., 2009). Previous studies have shown that blue light stimulating the hindpaws of *Mrgprd-ChR2* mice and *TRPV1-ChR2* mice drove paw lifting and licking behaviors, respectively (Beaudry et al., 2017; Warwick et al., 2021). However, whether reflexive behaviors or affective behaviors reliably reflect pain perception is not clear.

The spinal dorsal horn transmits and processes nocifensive information from nociceptors to brain (Sengul and Watson, 2015; Huang et al., 2019; Choi et al., 2020; Browne et al., 2021). Nocifensive information is processed by laminar organization *via* ascending projection neurons located in superficial (I–II_*o*_) and deeper (IV–V) lamina of the spinal cord (Wercberger and Basbaum, 2019). The superficial projection neurons to the thalamic and parabrachial nucleus contribute to the emotional responses to pain (Gauriau and Bernard, 2002). In contrast, the deeper projection neurons to reticular areas may transmit the motor reaction to external stimuli (Gauriau and Bernard, 2002). Exploring the underlying spinal substrates for the two dimensions of nocifensive behaviors evoked by selective activation of the Mrgprd^+^ and TRPV1^+^ subsets of nociceptors is essential for understanding their association with pain perception.

To elucidate the association of distinct nocifensive behaviors with pain, we first performed a detailed characterization of nocifensive behaviors and classified them as “reflexive” or “affective” responses following optogenetic activation of Mrgprd^+^ and TRPV1^+^ afferents. Afterward, we examined their capacity to produce aversion, an indication of “unpleasant” experience and real pain, under physiological and pathological conditions. Finally, we investigated the potential spinal substrates for transmitting the two dimensions of nocifensive behaviors. In summary, our data reveal distinct nocifensive behaviors evoked by selective activation of the Mrgprd^+^ and TRPV1^+^ subsets of nociceptors and the underlying spinal substrates.

## Materials and Methods

### Animals

All animal experiments were performed with protocols approved by the Animal Care and Use Committee of the University of Science and Technology of China. Mice were maintained under a 12 h light/dark cycle (lights on from 07:00 to 19:00) and with *ad libitum* access to water and food. *Mrgprd^CreERT2^*, *TPRV1^Cre^*, and *Ai32* (*Cre-dependent ROSA^ChR2–EYFP^*) mice were purchased from Jackson Laboratories. By crossing *Mrgprd^CreERT2^* mice or *TRPV1^Cre^* mice with *Ai32* mice, *Mrgprd^CreERT2^; Ai32* mice (referred to as *Mrgprd-ChR2* mice) and *TRPV1*^Cre^*; Ai32* mice (referred to as *TRPV1-ChR2* mice) were obtained. To label or manipulate adult *Mrgprd*-expressing nociceptors, five consecutive daily intraperitoneal injections of tamoxifen (100 μl, 10 mg/ml) starting at P21 in *Mrgprd^CreERT2^* and *Mrgprd-ChR2* mice were performed. Both males and females were included. Animals were assigned to different groups at random, and behavioral responses were measured in a blinded manner.

### Spared Nerve Injury

The detailed procedure of Spared Nerve Injury was described in previous study (Decosterd and Woolf, 2000). Briefly, animals were anesthetized with 3% isoflurane. Then, the left hindlimb was elevated and fixed in a lateral position. Using the femur as a landmark, the three peripheral branches (sural, common peroneal, and tibial nerves) of the sciatic nerves were exposed by blunt dissection of the overlying muscle. Both the tibial and common peroneal nerves were ligated with 4–0 silk and transected (1–2 mm section), while the sural nerve was carefully preserved. Then, the skin was sutured and disinfected with iodophor. In the sham group, the sciatic nerve was exposed but not ligated and severed. Mechanical and thermal sensitivity were used to assess the changes in pain threshold.

### Acute Light-Induced Nocifensive Behaviors

To study the behavioral responses evoked by optogenetic stimulation. Animals were individually placed in a chamber (6.5 cm × 6.5 cm × 6 cm) with a hollow floor of wire. A 1 mm diameter fiber (Inper, China) was connected to the laser (QAXK-LASER, China) to stimulate the glabrous skin of a hindpaw. The distance between the fiber tip and skin was 1 cm. Each animal received three trials (20 s optical stimulus per trial). In naïve *Mrgprd-ChR2* and *TRPV1-ChR2* mice, the light stimulation was applied to the left and right hindpaws alternately with a minimal 3 min interval. However, in sham/SNI *Mrgprd-ChR2* mice, the light stimulation was applied to left hindlimb (side of surgery) with a minimal 3 min interval. The following stimulus parameters were used: 2 Hz–1 mW/mm^2^, 2 Hz–10 mW/mm^2^, 2 Hz–20 mW/mm^2^, 5 Hz–1 mW/mm^2^, 5 Hz–10 mW/mm^2^, 5 Hz–20 mW/mm^2^, 10 Hz–1 mW/mm^2^, 10 Hz–10 mW/mm^2^, and 10 Hz–20 mW/mm^2^. Light intensity was measured by a laser power meter (LP1, Sanwa). The behavioral responses evoked by the optical stimulus were recorded by a camera (SONY HDR CX450). Lifting, holding, and fluttering responses were counted as reflexive behaviors, while jumping, licking, guarding and vocalization were counted as affective behaviors (Corder et al., 2017; Chamessian et al., 2019). The definition of the diverse action was as follows: Lift: Raise paws instead of moving around, Hold: Keep the paw raised for > 2 s, Flutter: Rapid and repeated lifts, Jump: All hindpaws are off the ground, Lick: Turn the head and lick the paw, Guard: Paw is lifted at the mid-body position or hold laterally, Vocalization: To make an audible sound, like a squeak.

### Real-Time Place Avoidance and Conditioned Place Avoidance

Mice were habituated in Real-Time Place Avoidance (RTPA) or Conditioned Place Avoidance (CPA) apparatus for two consecutive days (30 min per day) before the RTPA or CPA test. The apparatus (200 mm × 100 mm × 100 mm) consisted of two chambers (100 mm × 100 mm × 100 mm) with distinct stripe patterns. A small hole (40 mm × 40 mm) was reserved for the mice to move freely between the two chambers.

#### Real-Time Place Avoidance

Real-Time Place Avoidance test was designed based on previous research (Chamessian et al., 2019), which divided into three consecutive phases (pre-stimulation phase, stimulation phase, post-stimulation phase, 10 min per phase). During pre-stimulation phase, *Mrgprd-ChR2* mice or *TRPV1-ChR2* mice were allowed to explore freely between the two chambers. The preferred chamber was defined based on the time spent in each chamber before hindpaw transdermal light stimulation (1 cm away from the hindpaw). Then, optical stimulation (10 Hz, 20 ms pulse-width, 20 mW/mm^2^) was delivered during the stimulation phase. Blue light (473 nm) stimulation occurred when the mouse was in the preferred chamber, while yellow light (593.5 nm) was delivered when the mouse entered the paired chamber. Finally, the mouse explored the two chambers again without stimulation in the post-stimulation phase, and the time spent in the preferred chamber was recorded.

#### Conditioned Place Avoidance

Conditioned Place Avoidance experiment lasted for 4 days (10 min per day) and was divided into three phases (pre-stimulation phase, day 1; stimulation phase, day 2 and day 3; post-stimulation phase, day4). On day 1 and day 4, *Mrgprd-ChR2* mice or *TRPV1-ChR2* mice explored freely between the two chambers (10 min), and the time spent in the preferred chamber was recorded. On day 2 and day 3, mice received hindpaw transdermal light stimulation as RTPA test (blue light in the preferred chamber, yellow light in the paired chamber).

### Mechanical Hypersensitivity Test

Before mechanical hypersensitivity test, mice were placed on the perforated metal mesh floor and habituated in metal mesh containers (6.5 cm × 6.5 cm × 6 cm) for three consecutive “habituation” sessions (30 min per day). Then, the Dixon’s up-down method (Dixon, 1965) was used to evaluate the withdrawal threshold, which was measured by stimulating the plantar area of the hindpaws with a series of von Frey filaments with different strengths (g). Meanwhile, dynamic mechanical hypersensitivity was measured by brush (light stroking from heel to toe of the plantar area of hindpaws with a paintbrush), which are described in previous studies (Cheng et al., 2017; Zhang et al., 2018). The dynamic score was defined as following: scored 0, fast movement, lifting the stimulated paw for less than 1 s; scored 1, sustained lifting (more than 2 s) of the stimulated paw toward the body; scored 2, one strong lateral paw lift, above the level of the body or a startle-like jump; and scored 3: multiple flinching responses or licking of the affected paw. Three trials at 10 s intervals were performed, and an average score was noted for each mouse.

### Hargreaves Test

Thermal sensitivity was evaluated by a Hargreaves apparatus (Model390, IITC Life Science Inc., Woodland Hills, CA, United States). Mice were habituated for three consecutive days (60 min per day) in the clear arena on a glass with constant temperature (30°C). Then, the paw withdrawal latencies (PWL) were recorded when the plantar surface of the hindpaw was exposed to a beam of light (4 × 6 mm size, 25% of maximum intensity). Five trials were performed for each mouse with an interval of at least 5 min, and a cutoff of 20 seconds was used to prevent tissue damage. To minimize the variation, the maximum and minimum PWL trials were excluded, and the remaining three trials were used to calculate the average latency.

### Retrograde Labeling

To label spinal cord-lPBN projection neurons, Fluoro-gold (FG, Santa Cruz) was injected into lPBN. Briefly, the mouse head was fixed on a stereotaxic instrument (RWD, China). After exposing the skull, a hole was drilled for injection. Then, 600 nl of 1% FG in 0.9% normal saline was injected into the right lPBN (mediolateral (ML), –1.30 mm; anteroposterior (AP), –5.00 mm; dorsoventral (DV), –3.5 mm from bregma) using a glass pipette connected with the microinjection system (KDS). Seven days later, all mice were used for the c-Fos induction experiment.

### c-Fos Induction

To study optogenetics-induced c-Fos expression in the spinal cord and lateral parabrachial nucleus, background c-Fos fluorescence was minimized by placing mice in the training apparatus for a period of 4 h before treatment. Then, the hindpaw was stimulated for 10 min with blue light (10 Hz, 20 ms pulse-width, 20 mW/mm^2^). To investigate the baseline of c-Fos expression in the spinal cord, mice did not receive light stimulation after 4 h adaptation. Then, mice were allowed to move freely in the apparatus for an additional 1.5 h and sacrificed. The spinal cord and brain were excised for immunofluorescence (IF) analysis.

### Immunofluorescence

Mice were anesthetized by 3% isoflurane. Then, mice were intracardially perfused with 20 ml 0.01 M phosphate-buffered saline (PBS) (4°C) and 20 ml 4% paraformaldehyde (PFA) (4°C). After fixation with 4% PFA for an additional 12 h at 4°C and dehydration with 30% sucrose, tissues (brain, spinal cord, L3-L5, and DRG, L3-L5) were embedded in OCT (SAKURA, 4583) and sectioned (brain 30 μm, spinal cord 25 μm, DRG 15 μm) with a cryostat (Leica CM1950). Slides were permeabilized in 0.4% Triton X-100 (Sangon Biotech, A110694) (in PBS) for 15 min. Next, the slides were incubated in 10% normal goat serum (Absin, abs933) (in PBST) for 1 h to block the non-specific antibody binding. Then, slides were incubated with primary antibody (rabbit anti-c-Fos antibody, 1:1000, Synaptic Systems, Cat# 226003; rabbit anti-CGRP antibody, 1:1000, Sigma, Cat# C8198; Alexa-568-conjugated IB4, 1:1000, Thermo Fisher Scientific, Cat# I21412) for 2 h at room temperature. Next, after washing 3 times for 5 min in PBST, slides were incubated with secondary antibody (Alexa Fluor 594-AffiniPure Goat Anti-Rabbit IgG (H + L) antibody (1:500, Jackson, Cat# 111-585-003; Goat Anti-Rabbit IgG H&L (Alexa Fluor 647) preadsorbed antibody, 1:500, Abcam, Cat# ab150083) for 1 h at room temperature. After washing in PBS, the slides were sealed with anti-fluorescence quenching sealing tablets. Finally, images were recorded with Olympus confocal microscopes (FV3000, Olympus).

### *In situ* Hybridization

The detailed *in situ* hybridization (*ISH*) protocol was described in previous studies (Birren et al., 1993; Ma et al., 1998). The *Mrgprd*-probe labeled with digoxigenin was designed based on *the Allen Brain Map* website.^1^ Briefly, after designing specific primers, the *Mrgprd*-probe was amplified from the DRG-cDNA library. To avoid quenching autofluorescence, the EYFP signal was imaged before *ISH*. The *ISH* signals were images with transmitted light. The obtained *ISH* images were converted to pseudo-red and merged with EYFP by ImageJ software.

### Spinal Cord Slice Electrophysiology

#### Spinal Cord Slice Preparation

Parasagittal spinal cord slices with dorsal root and DRG attached were optimized based on previous studies (Cheng et al., 2017; Zhang et al., 2018). Mice (8–12 weeks) were anesthetized with pentobarbital sodium (2% w/v, i.p.) followed by intracardial perfusion of 25 ml ice-cold NMDG substituted artificial cerebrospinal fluid (NMDG-ACSF) containing (in mM) 93 N-methyl-d-glucamine (NMDG), 2.5 KCl, 1.2 NaH_2_PO_4_, 30 NaHCO_3_, 20 HEPES, 25 glucose, 2 thiourea, 5 Na-ascorbate, 3 Na-pyruvate, 0.5 CaCl_2_, 10 MgSO_4_ and 3 glutathione (GSH). The pH was titrated to 7.3–7.4 with HCl, and the osmolarity was 310–320 mOsm. The lumbar spinal cord was then removed to ice-cold oxygenated NMDG-ACSF, and the spinal cord with an attached dorsal root and DRG was cut using a vibratome (VT1200S, Leica). The slices were initially incubated in NMDG-ACSF for 10 min at 32°C and transferred to N-2-hydroxyethylpiperazine-N-2-ethanesulfonic acid ACSF (HEPES-ACSF) containing (in mM) 92 NaCl, 2.5 KCl, 1.2 NaH_2_PO_4_, 30 NaHCO_3_, 20 HEPES, 25 glucose, 2 thiourea, 5 Na-ascorbate, 3 Na-pyruvate, 2 CaCl_2_, 2 MgSO_4_ and 3 GSH (pH 7.3–7.4, 310–320 mOsm, oxygenated with 95% O_2_ and 5% CO_2_) for more than 1 h at 25°C. Slices were transferred to a recording chamber and perfused with standard ACSF continuously at 3–5 ml/min.

#### Patch-Clamp Recordings

Whole-cell recording experiments were performed as described previously (Cheng et al., 2017; Zhang et al., 2018). Recordings were made from randomly picked neurons in the lamina I–II_*o*_ and lamina II_*i*_ using oxygenated recording ACSF containing (in mM) 125 NaCl, 2.5 KCl, 2 CaCl_2_, 1 MgCl_2_, 1.25 NaH_2_PO_4_, 26 NaHCO_3_, 25 d-glucose, 1.3 sodium ascorbate and 3.0 sodium pyruvate, with pH at 7.3 and measured osmolality at 310–320 mOsm. The internal solution contains (in mM): 130 potassium gluconates, 5 KCl, 4 Na_2_ATP, 0.5 NaGTP, 20 HEPES, 0.5 EGTA, pH 7.28 with KOH, and measured osmolality at 310–320 mOsm. Data were acquired with pClamp 10.0 software using a MultiClamp 700B patch-clamp amplifier (Molecular Devices) and Digidata 1550B (Molecular Devices). Responses were low-pass filtered on-line at 2 kHz and digitized at 5 kHz. Afferents with the EYFP signal were visualized by fluorescence microscopy to determine the inner lamina II. Photostimulation was performed using a 473 nm laser (QAXK-LASER, China) and controlled using pClamp 10 software (Axon). To record light-evoked synaptic transmission, blue light at 20 ms duration was applied to dorsal horn at 10 s intervals. To record light-evoked excitatory postsynaptic currents (l-eEPSCs) or light-evoked inhibitory postsynaptic currents (l-eIPSCs), the membrane potential was held at –70 or –45 mV, respectively. To record light-evoked action potentials (l-eAPs), current-clamp recordings were performed at the resting membrane potential.

### Statistical Analysis

Data are expressed as the mean ± SEM. Statistical analyses were performed by GraphPad Prism 9. For comparison of two groups, data were subjected to Mann–Whitney *U* test or Student’s unpaired or paired *t*-test. For comparison of multiple groups, data were subjected to One-way repeated-measures ANOVA with holm-sidak test. For SNI-induced mechanical and thermal hypersensitivity changes, time-course measurements were assessed by Two-way repeated-measures ANOVA with holm-sidak test. *p* < 0.05 was considered as significant changes.

## Results

### Distinct Expression Profile of Channelrhodopsin-2 in Mrgprd^+^ and TRPV1^+^ Nociceptors

**FIGURE 1:**
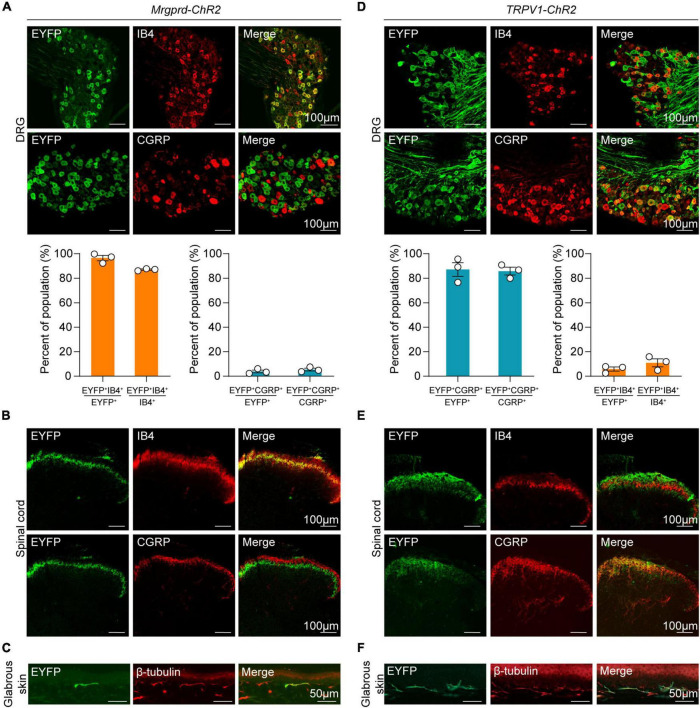
Distinct expression profile of ChR2 in Mrgprd^+^ and TRPV1^+^ nociceptors. **(A)** Co-localization of ChR2-EYFP (green) with Alexa-568-conjugated IB4 (red) or CGRP (red) (top) and statistical data for overlap quantification (bottom) in lumbar DRG from *Mrgprd-ChR2* mice (*n* = 3 animals per group). **(B)** Representative images of ChR2-EYFP (green) co-stained with IB4 (red) or CGRP (red) in lumbar spinal cord from *Mrgprd-ChR2* mice. **(C)** Representative images of ChR2-EYFP (green) co-stained with β-tubulin (red) in hindpaw glabrous skin from *Mrgprd-ChR2* mice. **(D)** Co-localization of ChR2-EYFP (green) with Alexa-568-conjugated IB4 (red) or CGRP (red) (top) and statistical data for overlap quantification (bottom) in lumbar DRG from *TRPV1-ChR2* mice (*n* = 3 animals per group). **(E)** Representative images of ChR2-EYFP (green) co-stained with IB4 (red) or CGRP (red) in lumbar spinal cord from *TRPV1-ChR2* mice. **(F)** Representative images of ChR2-EYFP (green) co-stained with β-tubulin (red) in hindpaw glabrous skin from *TRPV1-ChR2* mice. Scale bar = 100 μm for DRG and spinal cord sections, scale bar = 50 μm for skin sections.

To activate Mrgprd^+^ and TRPV1^+^ nociceptors in awake, freely moving animals, an optogenetic strategy was used. Here, we used an inducible *Mrgprd^CreERT2^* mouse line to manipulate this group of neurons in adult mice. We first crossed *Mrgprd^CreERT2^* mice with *Ai32* mice, resulting in double heterozygous *Mrgprd-ChR2* mice. Tamoxifen was then intraperitoneally injected at postnatal day 21 (P21-P25) in *Mrgprd-ChR2* mice, and analysis was performed at P42 when Mrgpra3^+^ and Mrgprb4^+^ neurons were rarely labeled so that we can specifically manipulate adult Mrgprd^+^ neurons (Olson et al., 2017; Ferrini et al., 2020). To examine the efficiency of Mrgprd^+^ neurons labeling by this strategy, *ISH* was performed on lumbar DRGs (L3-L5) of *Mrgprd-ChR2* mice. The overlapping of ChR2-EYFP and *Mrgprd* demonstrated that this strategy efficiently labeled Mrgprd^+^ neurons (Supplementary Figure 1). To examine the expression of ChR2-EYFP in non-peptidergic sensory neurons, we performed immunostaining for the non-peptidergic marker IB4 and the peptidergic marker CGRP (Zylka et al., 2005). In DRG, ChR2-EYFP^+^ neurons were colocalized with IB4 and almost non-overlapping with CGRP (Figure 1A, top). Quantitatively, ∼97% of ChR2-EYFP^+^ neurons were positive for IB4, and ChR2-EYFP^+^ neurons captured ∼89% of IB4^+^ DRG neurons, indicating that this inducible transgenic approach highly covered the non-peptidergic C-fiber subset (Figure 1A, bottom). In the spinal dorsal horn, the EYFP signals overlapped with IB4^+^ terminals in inner lamina II, but not with CGRP^+^ terminals, further indicating the specific expression of ChR2-EYFP in non-peptidergic neurons (Figure 1B). The feasibility of transdermal illumination to activate cutaneous Mrgprd^+^ nerve terminals was validated by the ChR2-EYFP signal observed in the glabrous skin epidermis of the plantar hindpaw and overlapped with the peripheral neural marker β-tubulin (Figure 1C).

Conversely, in *TPRV1-ChR2* mice, a line which expresses ChR2 in a group of non-overlapping peptidergic primary sensory neurons, over 85% of ChR2-EYFP^+^ neurons co-expressed CGRP, and EYFP^+^ neurons captured over 80% of CGPR^+^ DRG neurons (Figure 1D). Co-expression of IB4 signals was rarely detected in both DRG and spinal cord (Figures 1D,E). The ChR2-EYFP signal in the glabrous skin was also observed in *TRPV1-ChR2* mice (Figure 1F).

### Distinct Nocifensive Behaviors Following Optogenetic Activation of Cutaneous Mrgprd^+^ and TRPV1^+^ Fibers Under Normal Conditions

**FIGURE 2:**
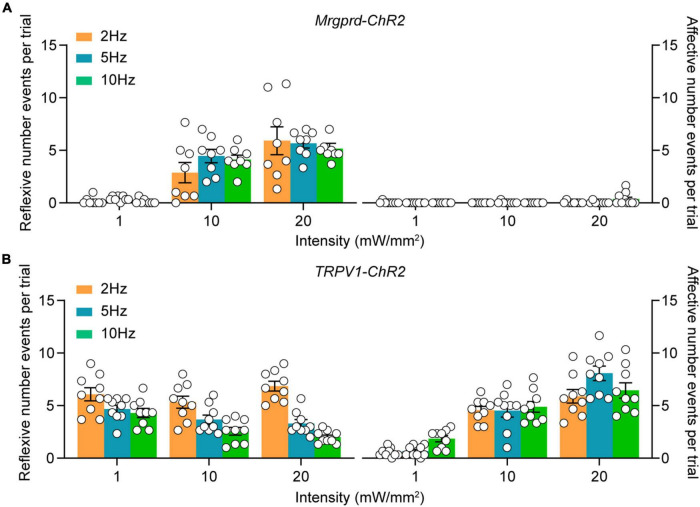
Characterization of cutaneous blue light-evoked nocifensive behaviors in naïve *Mrgprd-ChR2* and *TRPV1-ChR2* mice. **(A,B)** Reflexive and affective behaviors evoked by hindpaw plantar blue light stimulation at different intensities and frequencies in naïve *Mrgprd-ChR2* mice (*n* = 8 animals) **(A)** and *TRPV1-ChR2* mice (*n* = 9 animals) **(B)**. Data are presented as mean ± SEM.

To systematically characterize the nocifensive behaviors following different types of nociceptors activation, we carefully analyzed the behavioral responses in naïve *Mrgprd-ChR2* and *TRPV1-ChR2* mice following transdermal hindpaw photostimulation. To minimize neuron desensitization, three 20 s trials were conducted with at least 3 min between trials, alternating between the left and right hindpaws. To assess different facets of the behavioral responses, paw withdrawal like paw lifting, holding or fluttering were defined as “reflexive” behaviors while paw licking, guarding, jumping, rearing or vocalization were defined as “affective” behaviors in accordance with the paradigm used in previous studies (Chamessian et al., 2019; Corder et al., 2019). Interestingly, when exposed to blue light (473 nm) with a range of intensities (1, 10 and 20 mW/mm^2^) and pulse frequencies (2, 5, and 10 Hz), the behavioral profile of *Mrgprd-ChR2* mice was mostly reflexive but strikingly affective in addition to reflexive in *TPRV1-ChR2* mice (Figures 2A,B), revealing two distinct somatosensory processing pathways. Yellow (593.5 nm) light with the highest intensity exposure had no influence (Supplementary Figures 2A,B), excluding thermal or visual effects.

### The Correlation of Distinct Nocifensive Behaviors With Aversion Following Optogenetic Activation of Cutaneous Mrgprd^+^ and TRPV1^+^ Fibers Under Normal Conditions

**FIGURE 3:**
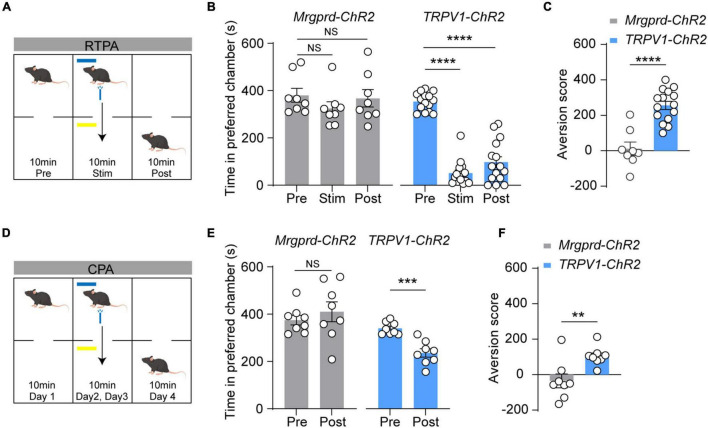
Blue light-induced RTPA and CPA in naïve *TRPV1-ChR2* mice but not naïve *Mrgprd-ChR2* mice. **(A)** Schematic drawing of RTPA experiment (see Methods). **(B,C)** Time spent in preferred chamber and aversion score of RTPA. Blue light stimulation induces strong aversion in naïve *TRPV1-ChR2* but not *Mrgprd-ChR2* mice for RTPA (*Mrgprd-ChR2*, *n* = 8 animals, *TRPV1-ChR2, n* = 15 animals). **(D)** Schematic drawing of CPA experiment (see Methods). **(E,F)** Time spent in preferred chamber and aversion score of CPA. Blue light stimulation induces strong aversion in naïve *TRPV1-ChR2* but not *Mrgprd-ChR2* mice for CPA. (*Mrgprd-ChR2*, *n* = 8 animals, *TRPV1-ChR2, n* = 8 animals). Data are presented as mean ± SEM. “NS,” no significance, **p < 0.01, ***p < 0.001, ****p < 0.0001. One-way repeated-measures ANOVA with holm-sidak test for **(B)**; paired Student’s two-tailed *t*-test for **(C,E)**; unpaired Student’s two-tailed *t*-test for **(F)**.

Aversion is commonly envisioned as a necessary feature and affective dimension of pain (Porreca and Navratilova, 2017; Rodriguez et al., 2017; Chamessian et al., 2019). To examine which type of nocifensive behavior is the readout of pain, a highly sensitive model for measuring aversive memory called two-chamber, RTPA assay was used (Figure 3A) (Chamessian et al., 2019). In naïve *Mrgprd-ChR2* mice, blue light stimulation cannot induce aversion, while *TRPV1-ChR2* mice showed strong aversion during the stimulation period as well as post-stimulation period (Figures 3B,C), indicating that reflexive behaviors mediated by Mrgprd^+^ fibers may not be a real-pain manifestation. Similarly, in a classic two-chamber avoidance assay called conditioned place avoidance (CPA) (Figure 3D) (Johansen et al., 2001), we found that optogenetic activation of TRPV1^+^ terminals but not Mrgprd^+^ terminals produced aversive memory of pain (Figures 3E,F).

### Optogenetic Activation of Cutaneous Mrgprd^+^ Fibers Induces Affective Behaviors and Aversion Under Neuropathic Pain Conditions

**FIGURE 4:**
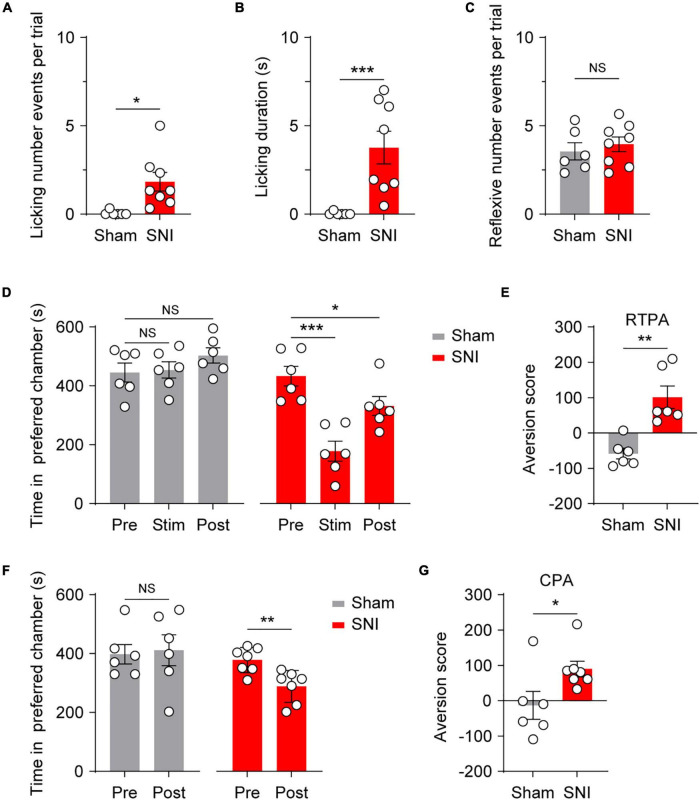
Optogenetic activation of cutaneous Mrgprd^+^ fibers induces affective behaviors and aversion under neuropathic pain conditions. **(A,B)** Statistical data for episodes of licking behaviors **(A)** and licking durations **(B)** evoked by optogenetic activation of Mrgprd^+^ fibers in sham *Mrgprd-ChR2* mice (*n* = 6 animals) and SNI *Mrgprd-ChR2* mice (*n* = 8 animals). **(C)** Statistical data for reflexive behaviors evoked by optogenetic activation of Mrgprd^+^ fibers in sham *Mrgprd-ChR2* mice (*n* = 6 animals) and SNI *Mrgprd-ChR2* mice (*n* = 8 animals). **(D–E)** Time spent in preferred chamber and aversion score for RTPA in sham/SNI *Mrgprd-ChR2* mice (*n* = 6 animals per group). **(F,G)** Time spent in preferred chamber and aversion score for CPA in sham/SNI *Mrgprd-ChR2* mice (sham, *n* = 6 animals, SNI, *n* = 7 animals). Data are presented as mean ± SEM. “NS,” no significance, *p < 0.05, **p < 0.01, ***p < 0.001. Mann–Whitney *U* Test for **(A,B,E,G)**; unpaired Student’s two-tailed *t*-test for **(C)**; One-way repeated-measures ANOVA with holm-sidak test for **(D)**; paired Student’s two-tailed *t*-test for **(F)**.

Given that optogenetic activation of adult cutaneous Mrgprd^+^ fibers produced reflexive behaviors without evoking aversion and, by extension, pain, we next examined whether activation of Mrgprd^+^ fibers can induce affective behaviors and aversion under pathological conditions. SNI was used as a relatively stable neuropathic pain model (Decosterd and Woolf, 2000; Bourquin et al., 2006) with filament-evoked static (Supplementary Figure 3A), brush-evoked dynamic (Supplementary Figure 3B) and thermal hypersensitivity (Supplementary Figure 3C). Strikingly, SNI *Mrgprd-ChR2* mice displayed a marked increase in affective behavioral responses number as well as time spent on licking in ipsilateral sides following hindpaw blue light simulation compared with sham *Mrgprd-ChR2* mice (Figures 4A,B), without changing reflexive events (Figure 4C). In this case, both RTPA and CPA can be induced (Figures 4D–G), further supporting the point that affective behaviors are tightly correlated with aversion and the reliable indication of pain.

### Distinct Spatial Distribution of c-Fos^+^ Neurons in the Spinal Cord Following Optogenetic Activation of Cutaneous Mrgprd^+^ and TRPV1^+^ Fibers Under Normal Conditions

**FIGURE 5:**
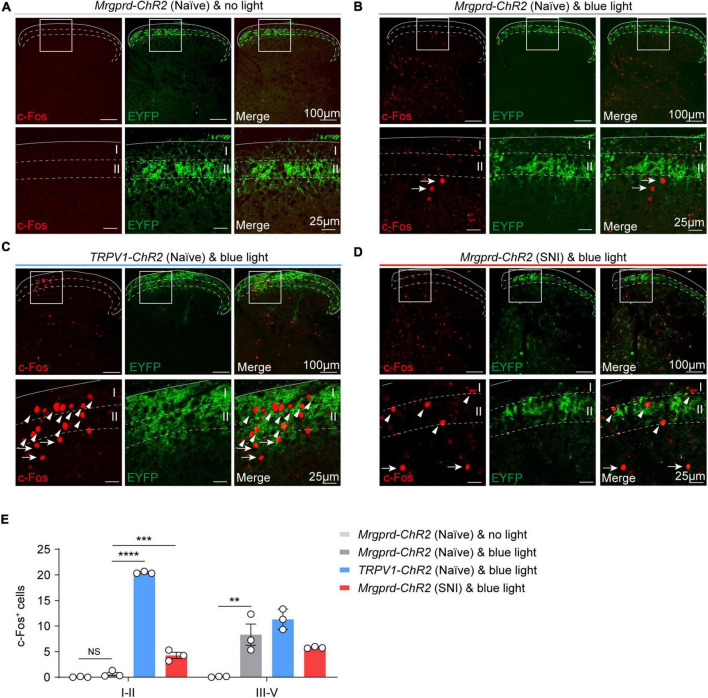
Spatial distribution of c-Fos^+^ neurons in the spinal cord following optogenetic activation of cutaneous Mrgprd^+^ and TRPV1^+^ fibers. **(A)** Representative images of c-Fos (red) baseline expression without light stimulation in *Mrgprd-ChR2* mice. **(B–D)** Representative images of c-Fos (red) expression within the spinal dorsal horn evoked by hindpaw blue light stimulation in naïve *Mrgprd-ChR2* mice **(A)**, naïve *TRPV1-ChR2* mice **(B)** and SNI *Mrgprd-ChR2* mice **(C)**. The arrowhead and arrow indicate c-Fos^+^ cells in lamina I–II and lamina III–V, respectively. Scale bar, 100 μm (25 μm for high magnification). **(E)** Statistical data of c-Fos^+^ cells within the lumbar spinal cord without light or evoked by hindpaw blue light stimulation in naïve *Mrgprd-ChR2* mice, naïve *TRPV1-ChR2* mice and SNI *Mrgprd-ChR2* mice (*n* = 3 animals per group). Data are presented as mean ± SEM. “NS,” no significance, **p < 0.001, ***p < 0.001, ****p < 0.0001. One-way repeated-measures ANOVA with holm-sidak test for **(E)**.

Classically, projection neurons (PNs) in superficial lamina (I–II_*o*_) and deeper lamina (IV–V) participate in signaling nociceptive specific information or wide dynamic range (innocuous to noxious ranges) information, respectively (Wercberger and Basbaum, 2019). To assess the spatial organization of spinal projection neurons for transmitting reflexive versus affective components in the dorsal spinal cord, we examined light-evoked activity marker c-Fos expression following 10 min of hindpaw blue light simulation in naïve *Mrgprd-ChR2* and *TRPV1-ChR2* mice. To avoid thermal activating c-Fos expression by sustained blue light illumination, a 1 cm distance away from hindpaw was conducted to obtain an optimal temperature at 25°C (Supplementary Figure 4). Without light stimulation, c-Fos is rarely expressed in the spinal cord in naïve *Mrgprd-ChR2* mice (Figures 5A,E). Intriguingly, with blue light stimulation, c-Fos^+^ neurons were observed and mainly distributed in the deep lamina (III–V) in *Mrgprd-ChR2* mice (Figures 5B,E), whereas a substantial amount of c-Fos^+^ neurons were observed in lamina I–II in *TRPV1-ChR2* mice (Figures 5C,E). Upon nerve injury, identical stimulation parameters also induced c-Fos expression in lamina I–II of the dorsal horn in *Mrgprd-ChR2* mice (Figures 5D,E).

### Optogenetic Activation of Cutaneous Mrgprd^+^ Fibers Induces c-Fos Expression on Superficial Spinal Cord-Lateral Parabrachial Nucleus Projection Neurons Under Neuropathic Pain Conditions

**FIGURE 6:**
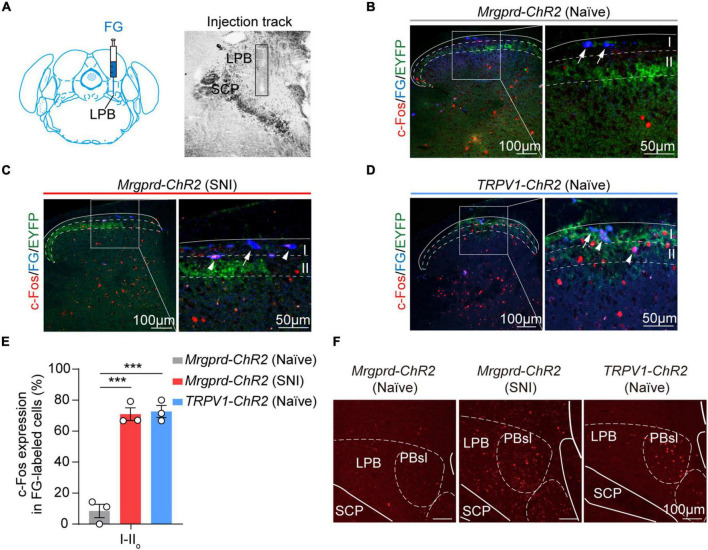
Optogenetic activation of cutaneous Mrgprd^+^ fibers induces c-Fos expression on superficial spinal cord-lPBN projection neurons under neuropathic pain conditions. **(A)** Schematic of Fluoro-gold injection site (left) and image of injection track within lPBN (right) (contralateral lPBN to SNI). **(B–D)** Representative images of colocalization of c-Fos^+^ neurons (red) with FG-labeled superficial lPBN-projection neurons (blue) in naïve *Mrgprd-ChR2* mice **(B)**, SNI *Mrgprd-ChR2* mice **(C)**, and naïve *TRPV1-ChR2* mice **(D)**. The arrow indicates FG^+^-labeled cells without c-Fos expression, and the arrowhead indicates double labeled (FG^+^ and c-Fos^+^) cells. **(E)** Statistical data for the percent of c-Fos^+^ neurons in FG-labeled cells (c-Fos^+^FG^+^/FG^+^) in lamina I–II_*o*_ of the spinal dorsal horn induced by blue light in naïve *Mrgprd-ChR2* mice, SNI *Mrgprd-ChR2* mice and naïve *TRPV1-ChR2* mice (*n* = 3 animals per group). **(F)** Representative images of c-Fos (red) expression in lPBN evoked by hindpaw blue light stimulation in naïve *Mrgprd-ChR2* mice, SNI *Mrgprd-ChR2* mice and naïve *TRPV1-ChR2* mice. Data are presented as mean ± SEM. ***p < 0.001. One-way repeated-measures ANOVA with holm-sidak test for **(E)**. Scale bar, 100 μm (50 μm for high magnification).

Given that the lPBN functions as a key relay station for processing somatosensory information, including pain (Mu et al., 2017; Rodriguez et al., 2017; Huang et al., 2019; Chiang et al., 2020; Choi et al., 2020; Browne et al., 2021), we next focused on spinal cord-lPBN projection neurons. To retrogradely label lPBN-projection neurons in the spinal cord, Fluoro-gold was injected into lPBN (Figure 6A). We observed that rare superficial projecting neurons were activated by Mrgprd^+^ afferents excitation under normal conditions (Figures 6B,E). However, superficial spinal cord-lPBN projection neurons largely overlapped with c-Fos^+^ neurons in SNI *Mrgprd-ChR2 mice*, somewhat similar to naïve *TRPV1-ChR2* mice (Figures 6C–E). Correspondingly, in naïve *Mrgprd-ChR2* mice, c-Fos was rarely induced in lPBN (Figure 6F). However, both in naïve *TRPV1-ChR2* mice and SNI *Mrgprd-ChR2* mice, lPBN showed significant c-Fos expression in response to hindpaw blue light simulation (Figure 6F). Moreover, c-Fos^+^ neurons were widely distributed in the superior lateral parabrachial nucleus (PBsl), a subregion of the lPBN conveying licking behavior associated with sustained pain (Huang et al., 2019). Taken together, these findings suggest that the superficial spinal cord-lPBN pathway possibly transmits affective responses. Nerve injury can “open” the superficial spinal cord-lPBN pathway recruited by cutaneous Mrgprd^+^ fibers.

### Synaptic Inputs and Outputs of Dorsal Horn Neurons From Mrgprd^+^ Nociceptors

**FIGURE 7:**
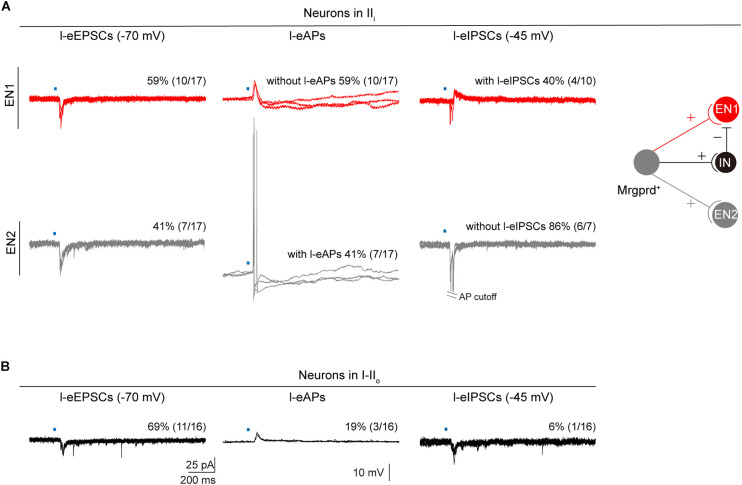
Synaptic inputs and outputs of dorsal horn neurons from Mrgprd^+^ nociceptors. **(A)** Left: Representative traces and percentage of neurons in lamina II_*i*_ with l-eEPSCs, l-eAPs and l-eIPSCs. Right: Schematic diagram for synaptic inputs and outputs of EN1 (red) and EN2 (gray) from Mrgprd^+^ nociceptors in naïve *Mrgprd-ChR2* mice. **(B)** Representative traces and percentage of neurons in lamina I–II_*o*_ with l-eEPSCs, l-eAPs, and l-eIPSCs in naïve *Mrgprd-ChR2* mice. (IN: inhibitory neurons, EN1: excitatory neurons that are gated by IN and receive excitatory inputs from Mrgprd^+^ nociceptors, EN2: excitatory neurons that are excited by Mrgprd^+^ nociceptors).

To further determine the underlying spinal substrates for nocifensive behaviors evoked by Mrgprd^+^ nociceptors, we explored synaptic inputs and outputs of dorsal horn neurons from Mrgprd^+^ nociceptors. To the end, we performed electrophysiological recordings in acute spinal cord slices prepared from naïve *Mrgprd-ChR2* mice. Given that Mrgprd^+^ nociceptors innervate lamina II_*i*_, we first examined l-eEPSCs and l-eAPs of lamina II_*i*_ neurons. By holding the membrane potential at −70 mV, we observed that 85% (17 of 20) of neurons in lamina II_*i*_ had detectable l-eEPSCs. However, among these 17 neurons, 59% (10 of 17) of neurons failed to fire APs. By holding the membrane potential at –45 mV, 40% (4 of 10) of neurons without l-eAPs showed l-eIPSCs (Figure 7A, top), indicating that this type of neurons is gated by feed-forward activation of an inhibitory neurons (IN). We defined these neurons without l-eAPs but with l-eIPSCs as type 1 excitatory neurons (EN1). In contrast, 41% (7 of 17) of neurons in lamina II_*i*_ had detectable l-eAPs, and 86% (6 of 7) of neurons with l-eAPs had no l-eIPSCs (Figure 7A, bottom). We defined these neurons with l-eAPs but without l-eIPSCs as type 2 excitatory neurons (EN2). According to the gate control theory of pain (Melzack and Wall, 1965), we proposed a model as following: EN1, EN2, and IN received excitatory inputs from the Mrgprd^+^ nociceptors. EN1 rather than EN2 is gated by inhibitory inputs from IN (Figure 7A, right). Therefore, the excitatory inputs from the Mrgprd^+^ nociceptors can activate EN2, but not EN1 (Figure 7A, right).

Next, we examined synaptic inputs and outputs of superficial lamina (I–II_*o*_) neurons where pain output neurons mostly located (Wercberger and Basbaum, 2019). 69% (11 of 16) of neurons in lamina I–II_*o*_ had detectable l-eEPSCs, but only 19% (3 of 16) of neurons can fire APs (Figure 7B), indicating that most of superficial projection neurons were “closed.” From above, our data further support that the Mrgprd^+^ nociceptors-deep spinal pathway possibly transmits reflexive behaviors and Mrgprd^+^ nociceptors-superficial spinal pathway which potentially transmits affective behaviors is “gated” under normal conditions.

## Discussion

**FIGURE 8:**
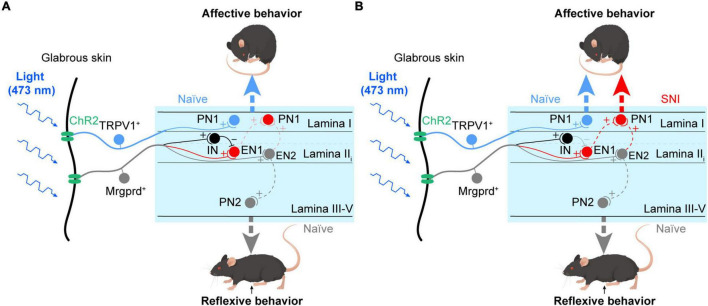
Parallel spinal pathways for transmitting reflexive and affective dimensions of nocifensive behaviors evoked by selective activation of the Mrgprd^+^ and TRPV1^+^ subsets of nociceptors. **(A)** Under normal conditions, activation of Mrgprd^+^ afferents elicits reflexive behaviors, which are possibly transmitted through the deep spinal pathway (gray, EN2-PN2). In contrast, activation of TRPV1^+^ afferents can elicit affective behaviors, which are possibly transmitted through the superficial spinal pathway (blue, TRPV1-PN1). **(B)** Under neuropathic pain conditions, activation of Mrgprd^+^ afferents can evoke affective behaviors by opening the superficial spinal pathway (red). (PN1: projection neurons in superficial layer, PN2: projection neurons in deeper layer, IN: inhibitory neurons, EN1: excitatory neurons that are gated by IN and receive excitatory inputs from Mrgprd^+^ nociceptors, EN2: excitatory neurons that are excited by Mrgprd^+^ nociceptors). We hypothesize that PN1 receives excitatory inputs from EN1 and EN2. The EN1-PN1 pathway which is “gated” *via* feed-forward activation of IN and the EN2-PN1 pathway which is “gated” *via* electric filtering of potassium currents transmit affective behaviors. After nerve injury, disinhibition of EN1 and/or central sensitization of PN1 cause “gate-opening” of superficial spinal pathway, leading to unmasking of affective behaviors evoked by Mrgprd^+^ nociceptors.

In the current work, taking advantage of *Mrgprd-ChR2* and *TRPV1-ChR2* mice, we revisited the reliability of reflexive and affective behaviors to assess pain sensation in rodents and explored the underlying spinal substrates for the two distinct nocifensive behaviors. The main findings are summarized below (Figure 8). Under physiological conditions, activation of Mrgprd^+^ afferents induces reflexive behaviors that are possibly transmitted through the deep spinal pathway (gray). In contrast, activation of TRPV1^+^ afferents can induce affective behaviors and aversion, which is possibly transmitted through the superficial spinal pathway (blue) (Figure 8A). Under neuropathic pain conditions, activation of Mrgprd^+^ afferents can elicit affective behaviors and aversion by opening the superficial spinal pathway (red) (Figure 8B). Altogether, we present here that affective behaviors and aversion may be good indicators of pain and reveal two parallel spinal pathways for transmitting the reflexive and affective dimensions of nocifensive behaviors (Figure 8).

### Conflict of Pain Assessment in Humans and Rodents

In humans, the withdrawal reflex can occur before the intensity of electrical stimuli reaches the pain threshold (Bromm and Treede, 1980). Imagining that when you step on the stone, you may feel no pain but still retract your feet, indicating the withdrawal reflex is not necessary for the readout of pain. However, when you step on the needle and sense intense pain, you will rapidly retract your feet and try to smooth the suffering, possibly by rubbing, blowing toward the wound and even groaning. The sequential behaviors are quite similar to jumping, licking and vocalization responses, which were defined as affective-motivational responses in rodents in previous work (Chamessian et al., 2019). Unfortunately, in rodents lacking self-report, people traditionally use the threshold eliciting withdrawal reflex to assess pain, leading to the dilemma that some effective analgesics on mice are useless on patients and potential effective drugs on patients are discarded.

### Correlation of Reflexive and Affective Behaviors With Pain

By using operant pain assays such as RTPA and CPA, we analyzed the correlation of reflexive and affective responses with aversion and, by extension, pain. Mrgprd^+^ nociceptors and TRPV1^+^ nociceptors, two subclasses of C-fibers, have been reported to mainly evoke lifting and licking responses following their activation, respectively (Zylka et al., 2005; Vrontou et al., 2013; Beaudry et al., 2017; Abdus-Saboor et al., 2019; Warwick et al., 2021). In our study, nocifensive behaviors evoked by optogenetic activation of Mrgprd^+^ and TRPV1^+^ afferents were characterized in detail. We defined paw lifting, holding, and fluttering as reflexive behaviors, while jumping, licking, guarding and vocalization as affective behaviors. Both RTPA and CPA were produced in *TRPV1-ChR2* mice rather than *Mrgprd-ChR2* mice. Combined with human studies showing that most mechanically sensitive C-fibers are activated by stimulus intensities that are reported as non-painful (Van Hees and Gybels, 1981; Handwerker et al., 1991), we conclude that affective behaviors rather than reflexive behaviors are reliable manifestations of pain. To further prove this viewpoint, we analyzed the behavioral responses in *Mrgprd-ChR2* mice under neuropathic pain conditions. In this case, affective behaviors and avoidance to blue light can be evoked.

### Parallel Spinal Pathways for Transmitting Reflexive and Affective Behaviors

Reflexive response and affective responses are two dimensions of pain. Parallel “pain” pathways for them can arise from different subpopulations of primary afferents (Braz et al., 2005; Huang et al., 2019) or different brain regions, such as the ventral posterolateral nucleus, thalamic complex and lateral parabrachial nucleus (Price, 2002; Han et al., 2015; Rodriguez et al., 2017; Huang et al., 2019). However, the laminar organization of the spinal cord neurons activated by the two classes of nociceptors is unclear. Here, by morphological and electrophysiological experiments, we demonstrated that the Mrgprd^+^ nociceptors-deep spinal cord pathway may transmit reflexive responses, while the TRPV1^+^ nociceptors-superficial spinal cord-lPBN pathway transmits affective responses and aversion under physiological conditions. This is quite similar to a previous study showing that functional connectivity between TRPV1^+^ nociceptors and spinal TAC1-expressing (TAC1^+^) excitatory neurons was required to elicit coping behaviors, while Mrgprd^+^ nociceptors were dispensible (Huang et al., 2019). After SNI, the superficial spinal cord-lPBN pathway was activated by Mrgprd^+^ nociceptors and transmitted affective responses and aversive behavior. Whether the linkage between Mrgprd^+^ nociceptors and spinal TAC1^+^ neurons is present and gated in naïve mice, but opened by SNI leading to unmasking of affective behaviors, is a puzzle.

### Mechanisms Underlying Gate Opening of Mrgprd^+^ Nociceptors-Superficial Pathway Under Neuropathic Pain Conditions

The distribution of activated neurons by Mrgprd^+^ nociceptors in superficial lamina can arise from aberrant sprouting of afferents (Woolf et al., 1992) or spinal neuronal network plasticity (Foster et al., 2015; Cheng et al., 2017; Dhandapani et al., 2018). After SNI, we did not observe any sprouting of Mrgprd^+^ afferents into the superficial region (Supplementary Figures 5A,B). The potential contribution of an increased number of ChR2-expressing neurons and an altered neuron population after SNI were also examined. We found that SNI did not change the density of ChR2-EYFP^+^ neurons or the proportion of ChR2-EYFP^+^ neurons expressing *Mrgprd* (Supplementary Figures 5C,D), which is consistent with a recent study (Warwick et al., 2021). We then focus on the network plasticity within the spinal cord. Our electrophysiological data identified that the Mrgprd^+^ nociceptors-superficial spinal cord pathway was “gated” under normal conditions. The gating mechanisms involve either classic feed-forward inhibition (Melzack and Wall, 1965) or electric filtering of subthreshold potassium currents (Zhang et al., 2018). After nerve injury, disinhibition is a critical contributor to the opened pathway from low-threshold mechanoreceptors to the superficial dorsal horn (Torsney and MacDermott, 2006). We speculate that the Mrgprd^+^ nociceptors-superficial spinal cord pathway is opened by the disinhibition of EN1 after SNI (Figure 8). This point is supported by a recent research that suggests a polysynaptic circuit occurs after SNI (Warwick et al., 2021). Nevertheless, whether central sensitization *via* attenuating potassium currents in PN1, which receives excitatory drive from EN2 (Figure 8), need to be studied in the future.

In summary, we identified two parallel spinal pathways for transmitting reflexive and affective dimensions of nocifensive behavior evoked by two different classes of nociceptors. By using aversive assay, we considered affective behaviors to be a better indicator of pain than reflexive behaviors. These findings remind us to use the rational behavioral assays to measure the pain sensation in rodents, which may promote discovery of effective analgesics. The spinal substrates for affective behaviors might also be a target for new analgesics.

## Data Availability Statement

The original contributions presented in the study are included in the article/Supplementary Material, further inquiries can be directed to the corresponding authors.

## Ethics Statement

The animal study was reviewed and approved by the Animal Care and Use Committee of the University of Science and Technology of China.

## Author Contributions

YZ and L-BW designed the project. X-JS generated electrophysiology data and contributed to their analysis. L-BW and X-JS performed the behavioral and immunofluorescence experiments. L-BW and Q-FW conducted *in situ* hybridization. X-YW and XX analyzed behavioral data. X-QL analyzed morphological data. MC injected Fluoro-gold. J-RY and AM managed the mouse colonies used in this study. WS and YZ supervised the project. YZ and L-BW wrote the first draft. All authors reviewed and edited the draft.

## Conflict of Interest

The authors declare that the research was conducted in the absence of any commercial or financial relationships that could be construed as a potential conflict of interest.

## Publisher’s Note

All claims expressed in this article are solely those of the authors and do not necessarily represent those of their affiliated organizations, or those of the publisher, the editors and the reviewers. Any product that may be evaluated in this article, or claim that may be made by its manufacturer, is not guaranteed or endorsed by the publisher.

## Footnotes

1
https://mousespinal.brain-map.org/imageseries/detail/100039689.html

## Supplementary Material

Supplementary Figure 1 The ChR2-EYFP^+^ neurons overlapped with *Mrgprd* (*ISH*). **(A)** Representative images of co-expression of ChR2-EYFP (green) with Mrgprd^+^ neurons identified by *in situ* hybridization (red) in DRG. Scale bar, 200 μm.

Supplementary Figure 2 Yellow light cannot evoke any nocifensive behaviors in naïve *Mrgprd-ChR2* mice or *TRPV1-ChR2* mice. **(A,B)** Nocifensive behaviors evoked by hindpaw plantar yellow light stimulation at 20 mW/mm^2^ with different frequencies (2, 5, 10 Hz) in naïve *Mrgprd-ChR2* mice (*n* = 6 animals) and *TRPV1-ChR2* mice (*n* = 6 animals). Data are presented as mean ± SEM.

Supplementary Figure 3 SNI induces mechanical and thermal hypersensitivity. **(A)** SNI-induced static allodynia to von Frey filaments (*n* = 7 animals per group). **(B)** SNI-induced dynamic allodynia to brush (*n* = 7 animals per group). **(C)** SNI-induced changes in withdrawal latency to heat (*n* = 7 animals per group). Data are presented as mean ± SEM. “NS,” no significance, **p* < 0.05, ^**^*p* < 0.01, ^***^*p* < 0.001, ^****^*p* < 0.0001. Two-way repeated-measures ANOVA with holm-sidak test for **(A–C)**.

Supplementary Figure 4 Temperature measurement following sustained blue light illumination. **(A)** Time course of blue light-induced changes in temperature following sustained blue light illumination (10 min, 10 Hz, 20 mW/mm^2^) with contact or 1 cm away from the meter.

Supplementary Figure 5 SNI did not change the distribution of Mrgprd^+^ afferents in the spinal cord, density of neurons expressing *Mrgprd* or proportion of ChR2-EYFP^+^ neurons expressing *Mrgprd*. **(A,B)** Representative images of ChR2-EYFP (green) distribution and colocalization with IB4 (red)/CGRP (blue) in the lumbar spinal cord in naïve *Mrgprd-ChR2* mice and SNI *Mrgprd-ChR2* mice. Scale bar, 100 μm. **(C)** Statistical data for the density of Mrgprd^+^ neurons identified by *in situ* hybridization in DRG sections from naïve *Mrgprd-ChR2* mice and SNI *Mrgprd-ChR2* mice (*n* = 3 animals per group). **(D)** Statistical data for the percent of Mrgprd^+^ cells that co-expressed with ChR2-EYFP (left) and the percent of ChR2-EYFP^+^ neurons that co-expressed with *Mrgprd* (right) in DRG from naïve *Mrgprd-ChR2* mice and SNI *Mrgprd-ChR2* mice (*n* = 3 animals per group). Data are presented as mean ± SEM. “NS,” no significance. Unpaired Student’s two-tailed *t*-test for **(C,D)**.

## Abbreviations

Mrgprd: Mas-related G protein-coupled receptor D
TRPV1: transient receptor potential vanilloid 1
ChR2: channelrhodopsin-2
SNI: spared nerve injury
lPBN: lateral parabrachial nucleus
IASP: the International Association for the Study of Pain
IB4: isolectin B4
CGRP: calcitonin gene-related peptide
RTPA: Real-time place avoidance
CPA: conditioned place avoidance
PWL: paw withdrawal latencies
FG: Fluoro-gold
PBS: phosphate-buffered saline
PFA: paraformaldehyde
OCT: optimal cutting temperature compound
IF: immunofluorescence
*ISH*: *in situ* hybridization
ANOVA: analysis of variance
SEM: standard error of mean
PNs: projection neurons
PBsl: superior lateral parabrachial nucleus
EPSCs: excitatory postsynaptic currents
APs: action potentials
IPSCs: inhibitory postsynaptic currents
IN: inhibitory neurons
EN: excitatory neurons
TAC1: tachykinin 1.
