# Supplementary material for: Parallel Spinal Pathways for Transmitting Reflexive and Affective Dimensions of Nocifensive Behaviors Evoked by Selective Activation of the Mas-Related G Protein-Coupled Receptor D-Positive and Transient Receptor Potential Vanilloid 1-Positive Subsets of Nociceptors
Source: Front Cell Neurosci. 2022 May 24;16:910670. doi: 10.3389/fncel.2022.910670 (PMC9175034; doi:10.3389/fncel.2022.910670)

Supplementary Figure 1. The ChR2-EYFP$^+$ neurons overlapped with *Mrgprd* (*ISH*).

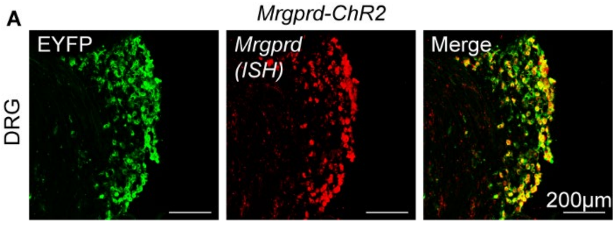

Supplementary Figure 2. Yellow light cannot evoke any nocifensive behaviors in naïve *Mrgprd-ChR2* or *TRPV1-ChR2* mice.

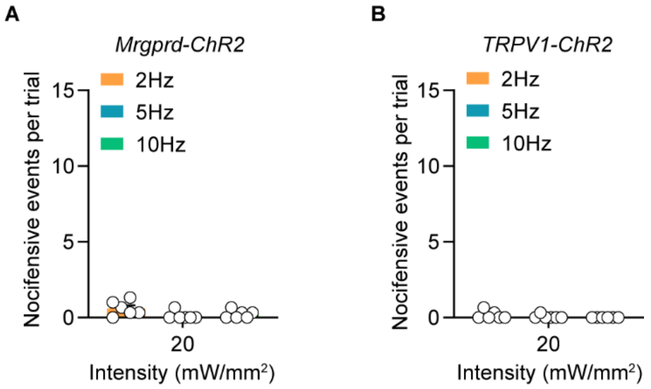

Supplementary Figure 3. SNI induces mechanical and thermal hypersensitivity.

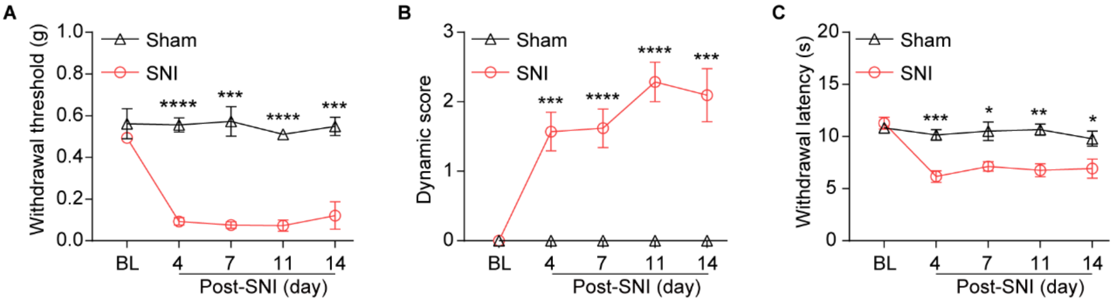

Supplementary Figure 4. Temperature measurement following sustained blue light illumination.

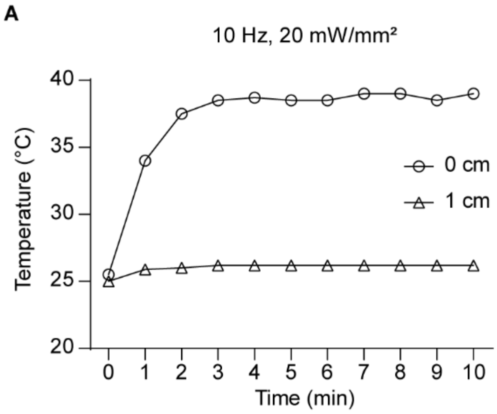

Supplementary Figure 5. SNI did not change the distribution of Mrgprd$^+$ afferents in the spinal cord, density of neurons expressing *Mrgprd* or proportion of ChR2-EYFP$^+$ neurons expressing *Mrgprd*.

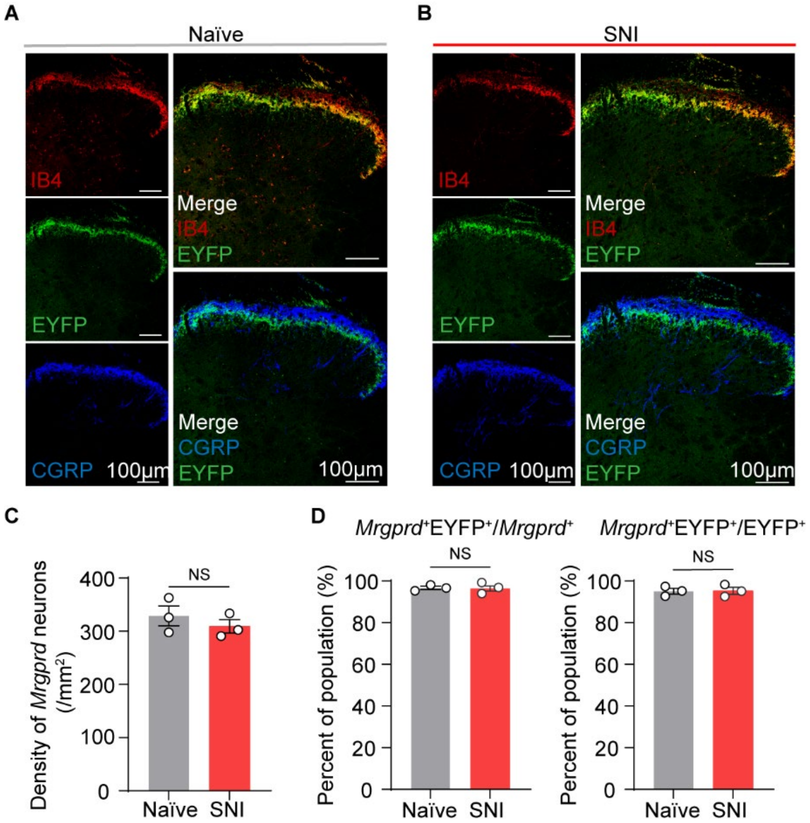

Supplement: Supplementary Figure 1 — The ChR2-EYFP+ neurons overlapped with Mrgprd (ISH). (A) Representative images of co-expression of ChR2-EYFP (green) with Mrgprd+ neurons identified by in situ hybridization (red) in DRG. Scale bar, 200 μm. [file Data_Sheet_1.PDF]
