# Supplementary material for: Parallel Spinal Pathways for Transmitting Reflexive and Affective Dimensions of Nocifensive Behaviors Evoked by Selective Activation of the Mas-Related G Protein-Coupled Receptor D-Positive and Transient Receptor Potential Vanilloid 1-Positive Subsets of Nociceptors
Source: Front Cell Neurosci. 2022 May 24;16:910670. doi: 10.3389/fncel.2022.910670 (PMC9175034; doi:10.3389/fncel.2022.910670)
Supplement: Supplementary file 2 [file Data_Sheet_2.docx]

**Supplementary Table S1. Antibodies used in this study.**

| Antibody | Manufacturer | Catalogue number | Dilution |
| --- | --- | --- | --- |
| anti-c-Fos | Synaptic Systems | 226003 | 1:1000 |
| anti-CGRP | Sigma | C8198 | 1:1000 |
| Alexa-568-conjugated IB4 | Thermo Fisher Scientific | I21412 | 1:1000 |
| anti-β-Tubulin | BioLegend | 657408 | 1:1000 |
| Alexa Fluor 594-AffiniPure Goat Anti-Rabbit IgG (H+L) antibody | Jackson ImmunoResearch Labs | 111-585-003 | 1:1000 |
| Goat Anti-Rabbit IgG H&L (Alexa Fluor 647) preadsorbed antibody | Abcam | ab150083 | 1：1000 |

**Supplementary Table S2. Sequences of the PCR primers used in the study.**

| Gene | Primer sequence |
| --- | --- |
| iNOS | FP; AATGCCCGTACCAGGCCCAAT  RP; TAGAGCCCACGCCATCCACTGG |
| IL-1β | FP; TTGTTCATCTCGGAGCCTGTA  RP; CTACTTCCTTTTCTTCCACGA |
| IL-6 | FP; GCACTAGGTTTGCCGAGTAGA  RP; GAGGAAGACACTGAGGTCGAA |
| TNF-α | FP; ATCCGCGACGTGGAACTAG  RP; AAGGTCTTGAGGTCCGCCA |
| IL-10 | FP; GCCTGGGGCATCACTTCTACC  RP; AAGGTCTTGAGGTCCGCCA |
| Arg-1 | FP; TCTTTGGCAGATATGCAGGGA  RP; AAGGTCTTGAGGTCCGCCA |
| UUR | FP; GGCAGAGCCAGGAAATTGC  RP; CACTATTAGGGAGAGGATTTGAACCT |
| COXI | FP; GCCCCAGATATAGCATTCCC  RP; GTTCATCCTGTTCCTGCTCC |
| COX3 | FP; CGTGAAGGAAACTACCCAGG  RP; CGCTCAGAAGAATCCTGCAA |
| ND1 | FP; GGATCCGAGCATCTTATCCA  RP; GGTGGTACTCCCTCTGTAAA |
| GAPDH | FP; AAGAAGGTGGTGAAGCAGG  RP; TGAGGGTGAGAAGGTGGAAG |
